# Distinct effects of psychiatric disorder diagnoses and severe emotional dysregulation on matrix metalloproteinase-9, proinflammatory cytokines, and inhibitory control function in adolescents with attention-deficit hyperactivity disorder or first-episode major affective disorders

**DOI:** 10.1093/ijnp/pyaf024

**Published:** 2025-04-12

**Authors:** Ju-Wei Hsu, Li-Chi Chen, Ya-Mei Bai, Shih-Jen Tsai, Mu-Hong Chen

**Affiliations:** Department of Psychiatry, Taipei Veterans General Hospital, Taipei, Taiwan; Department of Psychiatry, College of Medicine, National Yang Ming Chiao Tung University, Taipei, Taiwan; Department of Psychiatry, Taipei Veterans General Hospital, Taipei, Taiwan; Department of Psychiatry, College of Medicine, National Yang Ming Chiao Tung University, Taipei, Taiwan; Department of Psychiatry, General Cheng Hsin Hospital, Taipei, Taiwan; Department of Psychiatry, Taipei Veterans General Hospital, Taipei, Taiwan; Department of Psychiatry, College of Medicine, National Yang Ming Chiao Tung University, Taipei, Taiwan; Department of Psychiatry, Taipei Veterans General Hospital, Taipei, Taiwan; Department of Psychiatry, College of Medicine, National Yang Ming Chiao Tung University, Taipei, Taiwan; Department of Psychiatry, Taipei Veterans General Hospital, Taipei, Taiwan; Department of Psychiatry, College of Medicine, National Yang Ming Chiao Tung University, Taipei, Taiwan

**Keywords:** ADHD, first-episode bipolar disorder, major depressive disorder, matrix metalloproteinase-9, cytokines, inhibitory control function

## Abstract

**Background:**

Severe emotional dysregulation (SED) may represent an endophenotype of attention-deficit hyperactivity disorder (ADHD) and major affective disorders. However, the specific effects of SED and related psychiatric disorders, including ADHD, bipolar disorder (BD), and major depressive disorder (MDD), on matrix metalloproteinase-9 (MMP-9), proinflammatory cytokine levels, and inhibitory control function remain unclear.

**Methods:**

This study included 48 adolescents with ADHD, 39 with first-episode BD, 53 with first-episode MDD, and 46 healthy adolescents. SED was defined according to total *T* scores ≥210 on the Child Behavior Checklist Dysregulation Profile. Levels of MMP-9, interleukin (IL)-6, and C-reactive protein (CRP) were measured. Inhibitory control was assessed using the go/no-go task.

**Results:**

Generalized linear models adjusted for demographic and clinical data revealed significant main effects of diagnoses on MMP-9 (*P* = .009), CRP (*P* < .001), and IL-6 (*P* = .029) levels and on the standard deviation of mean response time on the go/no-go task (*P* = .004). A significant main effect of SED on MMP-9 levels (*P* = .048) was also observed. Adolescents with BD exhibited the highest MMP-9 and CRP levels and the poorest performance on the go/no-go task compared with the other groups. Adolescents with SED had significantly elevated MMP-9 levels than did those without SED.

**Discussion:**

Diagnoses of adolescent psychiatric disorder were associated with increased MMP-9, IL-6, and CRP levels and with inhibitory control dysfunction. In particular, SED was associated with elevated MMP-9 levels.

**Significance Statement**

The deficits in the blood–brain barrier (BBB) integrity, indicated by the increased matrix metalloproteinase-9 (MMP-9) levels, may play a crucial role in the pathomechanisms underlying bipolar disorder and severe emotional dysregulation. MMP-9 levels were further positively associated with C-reactive protein levels, suggesting a vicious cycle between BBB disintegrity and peripheral inflammation.

## INTRODUCTION

Growing evidence suggests that emotional dysregulation is a transdiagnostic endophenotype for major affective disorders, namely bipolar disorder (BD), major depressive disorder (MDD), and attention-deficit hyperactivity disorder (ADHD). This indicates that these psychiatric disorders may share pathomechanisms underlying emotional and cognitive regulation.^1,2^ Shaw et al. proposed a 3-component model of emotional dysregulation: (1) inappropriate and excessive emotional reaction relative to social norms, (2) uncontrolled and rapid shifts in emotions, and (3) abnormal attention allocation to emotional stimuli.^3^ Studies have indicated that severe emotional dysregulation (SED), as a poor clinical factor, was associated with increased risks of suicidality, diminished well-being, and long-term cognitive impairment.^1,2^

The role of matrix metalloproteinase-9 (MMP-9) in various psychopathologies, such as mania, depression, and ADHD, has garnered considerable scientific and clinical attention over the past decade.^4-6^ MMPs, including MMP-9, are critical regulators of the integrity of basement membranes lining blood vessels and play a pivotal role in cellular responses to their microenvironment, particularly in processes such as inflammation.^6^ Shibasaki et al. assessed serum MMP-9 levels in 21 patients with treatment-resistant depression and identified a positive correlation between depressive symptoms and MMP-9 levels.^7^ A cohort study of 681 adults, comprising 399 individuals with BD and 282 healthy controls, revealed that patients with BD exhibited significantly higher MMP-9 levels than did the control group even after adjustments for demographic variables, tobacco use, and obesity.^8^ Similarly, Kadziela-Olech et al. and Uzun Cicek et al. have reported that increased MMP-9 levels were correlated with the severity of ADHD symptoms (eg, inattention, hyperactivity, and opposition) and cognitive dysfunction (ie, errors in the Stroop test).^4,5^ However, most MMP-9-related studies on major affective disorders have focused on adult patients with recurrent episodes, limiting the generalizability of their findings to adolescents experiencing the first episode. Additionally, no study has explored the potential association between elevated MMP-9 levels and emotional dysregulation, despite both conditions being prevalent in major affective disorders and ADHD. Our study was the first to elucidate the role of MMP-9 in the SED.

Immunophenotypes, including increased levels of proinflammatory cytokines, have been reported in both major affective disorders and ADHD in recent decades.^9-11^ A meta-analysis study of 1046 patients with ADHD and 3333 healthy controls demonstrated that patients with ADHD exhibited significantly higher levels of interleukin (IL)-6, but not C-reactive protein (CRP), than did the controls.^9^ Maayan et al. also supported the causal role of IL-6 in the development of depression.^10^ In a study involving 35 adolescents with first-episode BD, 29 with first-episode MDD, and 22 healthy controls, patients with BD exhibited the highest levels of CRP (*P* = .023) and IL-6 (*P* = .022), regardless of the severity of clinical symptoms.^11^ Only a few studies have assessed the association between proinflammatory cytokines and emotional dysregulation.^12^ Holtmann et al. measured CRP levels and the Child Behavior Checklist Dysregulation Profile (CBCL-DP) among 133 children and adolescents.^12^ They reported an association between elevated CRP levels and higher total CBCL-DP scores; their findings suggest the crucial role of CRP in the pathomechanism underlying emotional dysregulation.^12^ Furthermore, Power et al. indicated a significant association between emotional dysregulation and higher CRP levels, regardless of body mass index (BMI) MDD diagnosis.^13^

Inhibitory control dysfunction is a core cognitive deficit in ADHD and is commonly reported among patients with major affective disorders.^14,15^ A meta-analysis of inhibitory control performance in 13 807 participants with various mental disorders revealed that patients with BD exhibited the most pronounced inhibitory control deficits, indicated by an increased number of errors, followed by those with ADHD and MDD.^15^ Alvarez et al. examined the associations between proinflammatory cytokines and performance on the go/no-go task in a large cohort of 30 528 individuals and identified an association between IL-6 reactivity and changes in task accuracy.^16^ Similarly, a cross-sectional study of 105 adolescents reported that BMI, an index of systemic low-grade inflammation, and the proinflammatory cytokine fibrinogen were negatively associated with inhibitory control function.^17^ Despite these findings, the potential association between MMP-9 levels and inhibitory control dysfunction has yet to be explored.

To address the aforementioned research gaps, we measured MMP-9, CRP, and IL-6 levels and assessed inhibitory control function by using the go/no-go task among adolescents with ADHD and those with major affective disorders, namely BD and MDD. Because SED is conceptualized as a transdiagnostic endophenotype among ADHD and major affective disorders, we investigated the effects of both diagnoses (ADHD and major affective disorders) and SED on MMP-9, CRP, and IL-6 levels and on inhibitory control function. We hypothesized that adolescents in the 3 diagnostic groups would exhibit higher levels of MMP-9 and proinflammatory cytokines and poorer performance in the go/no-go task compared with healthy controls. Additionally, we posited that SED would be associated with increased levels of MMP-9, CRP, and IL-6 and with inhibitory control dysfunction, regardless of the diagnostic classification.

## METHODS

### Participants and Study Procedure

In the current study, we recruited adolescents aged 12–17 years who had a diagnosis of ADHD or first-episode major affective disorders, namely BD and MDD. Additionally, the current study excluded patients with a comorbid condition of major affective disorders and ADHD. In order to avoid the confounding effect of severe mood symptoms, we only included adolescents who scored ≤18 of the total scores on the 17-item Hamilton Rating Scale for Depression (HDRS) and <10 of the total scores on the Young Mania Rating Scale (YMRS).^18,19^ We excluded participants with a lifetime diagnosis of schizophrenia, organic mental disorders, alcohol and substance use disorders, or cigarette smoking, as well as those with a prior history of significant physical illnesses like epilepsy, autoimmune disorders, or neurovascular diseases. The Swanson, Nolan, and Pelham-Version IV (SNAP-4) and the CBCL-DP were completed by adolescents’ parents.^20^ The SED was defined based on the total *T* scores of the CBCL-DP ≥210 (over 2 standard deviations higher than the mean for the anxiety/depression, aggression, and attention subscales of the CBCL).^20,21^ In addition, we recruited a group of healthy controls who had no prior history of the physical or mental illnesses listed earlier. All procedures in this study complied with the ethical standards of the national and institutional committees governing human experimentation, following the 1975 Helsinki Declaration and its 2008 revision. The Institutional Review Boards of Taipei Veterans General Hospital approved all protocols involving human subjects. Written informed consent was obtained from all participants and the parents or guardians of adolescent subjects.

### Assessment of CRP, IL-6, and MMP-9 Levels

We measured CRP, IL-6, and MMP-9 levels in each participant using enzyme-linked immunosorbent assay (ELISA) kits from R&D Systems. Fasting serum samples were collected in serum separator tubes, allowed to clot for 30 minutes, and collected between 9:00 am and 12:00 pm. All samples were subsequently stored at −80 °C until analysis. Assays were conducted in accordance with the manufacturer’s instructions. The final absorbance was measured at 450 nm using an ELISA plate reader (Bio-Tek Power Wave Xs) and analyzed with Bio-Tek’s KC Junior software (Winooski). The standard range was set according to the manufacturer’s guidelines, and a linear regression *R*² value of ≥0.95 was considered indicative of a reliable standard curve.

### Assessment of Inhibitory Control Function

The go/no-go task was used to measure inhibitory control function. Participants were instructed to react promptly by pressing a key when the × symbol appeared and to refrain from responding when the + symbol was shown. Following a pretest where all answers were correct, participants undertook the formal test, during which their errors and reaction times (mean ± standard deviation) were recorded. The go/no-go task has been commonly used in our previous studies.^22,23^

### Statistical Analysis

For between-group comparisons, continuous variables were analyzed using the *F*-test and categorical variables using the χ^2^ statistic. We employed generalized linear models (GLMs), adjusting for demographic characteristics (age, sex, and BMI) and clinical symptoms (HDRS, YMRS, and SNAP-4), to examine the main effects of diagnoses and SED, and their interactive effects on inhibitory control function. Following these adjustments, additional GLMs with a gamma log link were conducted to evaluate the main effects and interaction of diagnoses and SED on CRP, IL-6, and MMP-9 levels. Statistically significant effects from the GLMs were further explored using post-hoc analyses. Furthermore, we used the GLMs with a gamma log link, adjusting for demographic characteristics, diagnoses, SED, and clinical symptoms, to assess associations of inhibitory control function with MMP-9 levels, as well as CRP and IL-6 levels. Finally, we performed the GLMs with a gamma log link to investigate the associations of MMP-9 levels with CRP and IL-6 levels. A 2-tailed *P*-value of less than .05 was considered statistically significant. All data processing and statistical analyses were performed using SPSS software, version 17 (SPSS Inc.).

### Data Availability

The datasets collected and/or analyzed during the current study are available from the corresponding author upon reasonable request. However, they are not publicly accessible due to ethical regulations governing clinical trials in Taiwan.

## RESULTS

In all, 48 adolescents with ADHD, 39 adolescents with first-episode BD, 53 adolescents with first-episode MDD, and 46 healthy adolescents were enrolled in the present study, with a mean age of approximately 16 years (Table 1). Of those with ADHD or major affective disorders, 11 (23.4%) adolescents with ADHD, 9 (23.7%) adolescents with BD, and 12 (25.5%) with MDD met the SED definition (*P* > .05) (Table 1). Adolescents with first-episode BD or MDD had higher total HDRS (*P* < .001) and YMRS (*P* < .001) scores than did those with ADHD and healthy controls (Table 1). Adolescents with ADHD exhibited the highest total SNAP-4 scores (*P* < .001) compared with the other 3 groups (Table 1). In addition, of 140 adolescents with ADHD or major affective disorders and 46 healthy adolescents, 32 adolescents were classified as the SED group and 154 as the non-SED group (Table 1). The SED group had higher total HDRS (*P* < .001), YMRS (*P* = .002), and SNAP-4 (*P* < .001) scores than did the non-SED group (Table 1).

**Table 1.** Demographic and clinical characteristics between groups.

	Patient group (*n* = 140)						SED (−)	SED (+)	
	A. ADHD (*n* = 48)	B. BD (*n* = 39)	C. MDD (*n* = 53)	D. HC (*n* = 46)	*P*-value	Post-hoc	(*n* = 154)	(*n* = 32)	*P*-value
Age (years, SD)	15.54 (1.37)	16.31 (1.30)	15.83 (1.82)	16.13 (1.38)	.084		15.89 (1.47)	15.75 (1.48)	.630
Sex (*n*, %)					<.001				.052
Male	30 (62.5)	11 (28.2)	14 (26.4)	20 (43.5)			67 (43.5)	8 (25.0)	
Female	18 (37.5)	28 (71.8)	39 (73.6)	26 (56.5)			87 (56.5)	24 (75.0)	
BMI (SD)	23.66 (6.17)	24.17 (5.78)	21.46 (3.98)	21.54 (3.63)	.014	A~B > C~D	22.06 (4.82)	24.92 (5.85)	.004
Family income (SD, USD)	2858.32 (2143.44)	4052.25 (7826.14)	3816.59 (4183.40)	3149.60 (2138.61)	.630		3771.34 (4979.43)	2564.12 (2744.80)	.197
Clinical symptoms (SD)									
HDRS	5.46 (4.86)	10.77 (4.90)	12.53 (4.22)	0.46 (0.86)	<.001	C > B > A > D	6.08 (5.84)	11.88 (5.21)	<.001
YMRS	2.10 (0.99)	3.51 (1.88)	2.68 (0.85)	0.07 (0.25)	<.001	B > C > A > D	1.85 (1.85)	2.88 (2.88)	.002
SNAP-4	34.21 (13.09)	21.57 (14.49)	21.10 (12.51)	9.56 (7.45)	<.001	A > B~C > D	18.58 (18.58)	35.66 (35.66)	<.001
CBCL-DP, *T* scores	195.38 (22.66)	185.24 (44.80)	194.87 (24.57)	155.00 (8.24)	<.001	A~B~C > D	173.54 (26.43)	225.34 (14.99)	<.001
Diagnosis (*n*, %)									
ADHD	-	-	-	-			37 (24.0)	11 (34.4)	
BD	-	-	-	-			30 (19.4)	9 (28.1)	
MDD	-	-	-	-			41 (26.6)	12 (37.5)	
HC	-	-	-	-			46 (30.0)	-	
SED (*n*, %)	11 (23.4)	9 (23.7)	12 (25.5)	0 (0.0)	.004	A~B~C > D	-	-	
Medications (*n*, %)									
ADHD medications	25 (52.1)	7 (17.9)	4 (7.5)	0 (0.0)	<.001	A > B~C > D	26 (18.1)	9 (28.1)	.222
Antidepressants	6 (12.5)	18 (46.2)	37 (69.8)	0 (0.0)	<.001	C > B > A > D	42 (29.2)	14 (43.8)	.141
Mood stabilizers	0 (0.0)	12 (30.8)	1 (1.9)	0 (0.0)	<.001	B > C~A~D	9 (6.3)	4 (12.5)	.258
Atypical antipsychotics	7 (14.6)	26 (66.7)	23 (43.4)	0 (0.0)	<.001	B > C > A > D	38 (26.4)	16 (50.0)	.011
Benzodiazepines or Z-drugs	6 (12.5)	16 (41.0)	23 (43.4)	0 (0.0)	<.001	B~C > A > D	30 (20.8)	13 (40.6)	.024

Abbreviations: ADHD, attention-deficit hyperactivity disorder; BD, bipolar disorder; BMI, body mass index; CBCL-DP, Child Behavior Checklist Dysregulation Profile; HC, healthy control; SD, standard deviation; SED, severe emotional dysregulation; HDRS, 17-item Hamilton Rating Scale for Depression; MDD, major depressive disorder; SNAP-4, Swanson, Nolan, and Pelham-Version IV; USD, United States dollars (1 USD is equal to 32.395 new Taiwan dollars); YMRS, Young Mania Rating Scale.

**Figure 1.**
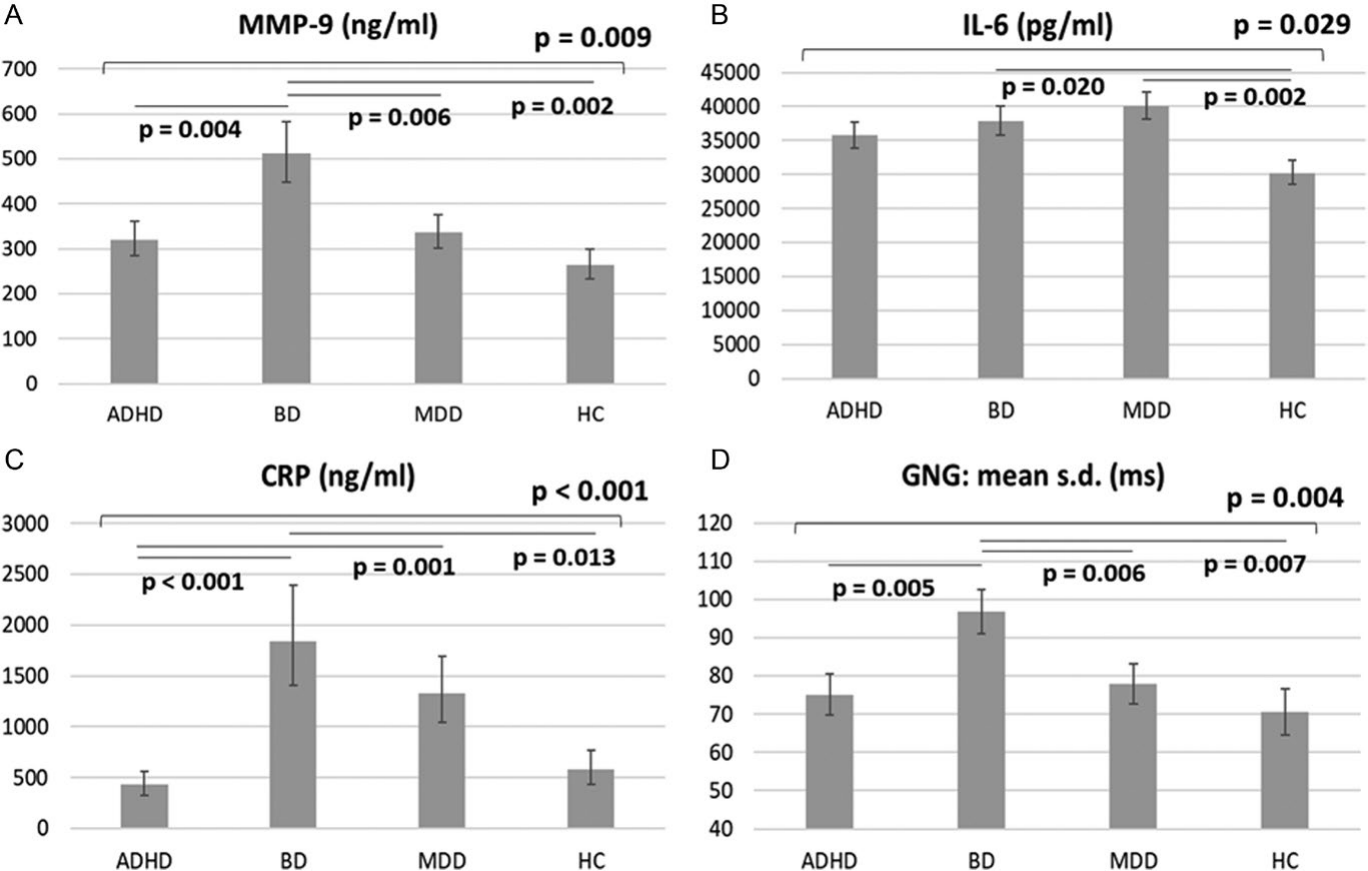
Estimated biomarker levels and cognitive function between groups using the generalized linear models with adjustment of demographic data, BMI, SED condition, and clinical symptoms. (A–C) with gamma log link and (D) with linear. ADHD, attention-deficit hyperactivity disorder; BD, bipolar disorder; CRP, C-reactive protein; GNG, go/no-go task; HC, healthy control; IL, interleukin; MDD, major depressive disorder; MMP-9, matrix metalloproteinase-9; SD: standard deviation; SED, severe emotional dysregulation.

**Figure 2.**
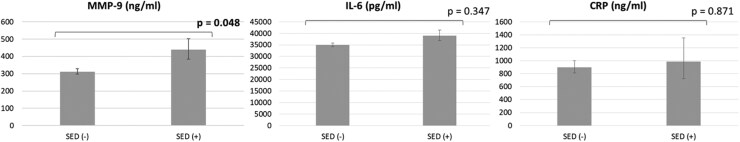
Estimated MMP-9, CRP, and IL-6 levels between SED groups using the generalized linear model with adjustment of demographic data, BMI, diagnoses, and clinical symptoms. IL, interleukin; MMP-9, matrix metalloproteinase-9; SED, severe emotional dysregulation.

The GLMs with a gamma log link, adjusting for demographic characteristics and clinical symptoms, found the main effects of diagnoses on MMP-9 (*P* = .009), CRP (*P* < .001), and IL-6 (*P* = .029) levels and also showed a main effect of SED (*P* = .048) on MMP-9 levels (Table 2). In addition, the GLMs identified a main effect of diagnoses (*P* = .004) on the standard deviation of the mean time on the go/no-go task (Table 2). The post-hoc analyses showed that adolescents with BD exhibited the highest MMP-9 levels (*P* = .009) compared with the other 3 groups (Figure 1). IL-6 levels were significantly higher in adolescents with BD (*P* = .020) or MDD (*P* = .002) than were the control group (Figure 1). Adolescents with ADHD exhibited nonsignificantly higher IL-6 levels (*P* = .052) than did the healthy controls (Figure 1). Adolescents with BD (*P* < .001) or MDD (*P* = .001) had significantly higher CRP levels than those with ADHD (Figure 1). CRP levels did not differ (*P* > .05) between adolescents with ADHD and the control group (Figure 1). Adolescents with BD exhibited the worst inhibitory control function (*P* = .004), indicated by the standard deviation of the mean time on the go/no-go task, compared with the other 3 groups (Figure 1). Figure 2 demonstrated that adolescents with SED had increased levels of MMP-9 (*P* = .048) compared with those without SED after adjusting for demographic characteristics and clinical symptoms.

**Table 2.** Generalized linear models with adjustment of age, sex, BMI, and clinical symptoms (HDRS, YMRS, and SNAP-4) between groups.

	Wald χ2	*P*-value	Wald χ2	*P*-value	Wald χ2	*P*-value
	MMP-9		CRP		IL-6	
Diagnosis (D)	11.63	**.009**	20.43	**<.001**	9.00	**.029**
SED (S)	3.90	**.048**	0.03	.871	0.96	.327
D*S	7.32	**.026**	1.02	.602	0.81	.668
	GNG: errors		GNG: mean time		GNG: mean time SD	
Diagnosis (D)	4.86	.182	3.57	.311	13.09	**.004**
SED (S)	0.78	.376	0.07	.788	0.46	.498
D*S	9.69	**.008**	1.11	.573	12.63	**.002**

Abbreviations: BMI, body mass index; CRP, C-reactive protein; GNG, go/no-go task; HDRS, 17-item Hamilton Rating Scale for Depression; IL, interleukin; MMP-9, matrix metalloproteinase-9; SD, standard deviation; SED, severe emotional dysregulation; SNAP-4, Swanson, Nolan, and Pelham-Version IV; YMRS, Young Mania Rating Scale.

Bold type indicates the statistical significance (*P* < .05).

Additionally, we found no associations between inhibitory control function and MMP-9 levels, as well as CRP and IL-6 levels (all *P* > .05). Finally, we found that MMP-9 levels were positively associated with CRP levels (B = 0.002, *P* < .001) but not IL-6 levels (*P* = .389).

## DISCUSSION

Our findings partially supported the study hypotheses, particularly in demonstrating that adolescents with first-episode BD exhibited the highest levels of MMP-9 and CRP and the poorest performance on the go/no-go task. Both adolescents with first-episode BD and those with first-episode MDD exhibited significantly elevated IL-6 levels compared with the control group, whereas adolescents with ADHD only exhibited a trend of increased IL-6 levels compared with the control group. CRP levels were significantly higher among adolescents with major affective disorders than among those with ADHD and healthy controls. Notably, SED was associated with only increased MMP-9 levels but not with elevated CRP or IL-6 levels or with impairments in inhibitory control function.

The most crucial finding of this study was that adolescents with first-episode BD, but not those with first-episode MDD or ADHD, exhibited elevated MMP-9 levels. This suggests that blood–brain barrier dysfunction occurs in the early stages (first episode) of BD but not in the early stages of MDD.^24^ Dickerson et al. assessed MMP-9 levels in a cohort of 399 adults with BD (mean age, 34.1 ± 13.0 years) and 282 age-matched controls and revealed that individuals with BD exhibited significantly elevated MMP-9 levels compared with the control group.^8^ Additionally, they found no association between MMP-9 levels and cognitive functioning, as evaluated using the Repeatable Battery for the Assessment of Neuropsychological Status.^8^ An exploratory biomarker study of 96 adult patients with BD (mean illness duration: 10 years) and 115 healthy controls identified MMP-9, but not other proteomics-identified upregulated proteins such as peroxiredoxin 2 and carbonic anhydrase 1, as a candidate biomarker of BD.^25^ The present study is the first to confirm increased MMP-9 levels among adolescents with BD; however, we did not observe an association between MMP-9 levels and inhibitory control function.

Our study is the first to report that patients with SED exhibited elevated MMP-9 levels compared with those without SED, regardless of psychiatric diagnoses or clinical symptoms. Preclinical research has highlighted the crucial role of MMP-9 in various brain regions involved in emotional regulation, including the hippocampus, anterior cingulate cortex, and prefrontal cortex.^26,27^ In a study of transgenic mice with redox dysregulation due to a permanent deficit in glutathione synthesis, Dwir et al. demonstrated that the oxidative stress-induced redox-sensitive MMP-9 activation in the anterior cingulate cortex; this activation triggered a cascade of nuclear factor-kB activation and the secretion of various proinflammatory cytokines.^27^ Furthermore, Dwir et al. suggested a detrimental role of MMP-6 in the vicious cycle of oxidative stress and neuroinflammation.^27^ An animal study using a corticosterone-induced mouse model of depression and anxiety revealed increased MMP-9 levels in the prefrontal cortex and hippocampus.^26^ The findings of these animal studies, which indicated that MMP-9 altered the functioning of brain regions associated with emotion regulation, may confirm our study’s finding that SED was associated with elevated MMP-9 levels. Additionally, we observed a positive relationship between MMP-9 levels and CRP levels, further supporting the role of MMP-9 in the inflammatory process.

Our findings of elevated MMP-9 levels in individuals with BD or SED may echo evidence suggesting that MMP-9 could be a potential therapeutic target in neuropsychiatry.^28^ MMP-9 plays a crucial role in the plasticity of excitatory synapses, influencing synaptic remodeling and neural connectivity.^28,29^ Rybakowski et al. identified the involvement of the MMP-9 gene in prefrontal cognitive functions, including executive function and inhibitory control, which are particularly linked to emotional regulation and high-level cognitive control.^30^ However, although several MMP-9 inhibitors, such as ilomastat (GM6001) and prinomastat (AG3340), have been developed, none of them have been investigated for the treatment of mental disorders yet.

Evidence suggests that BD is a more inflammatory condition than MDD.^31,32^ Our findings further support this, indicating higher CRP levels in BD, compared with MDD, even in the first episode. This is consistent with the findings of Wu et al., who indicated that elevated CRP levels were associated with diagnostic progression from MDD to BD in adolescents.^33^ In terms of CRP levels, our results also suggest that ADHD was less inflammatory than the 2 major affective disorders, which is consistent with the findings of Misiak et al., who reported no significant association between ADHD and CRP levels.^9^ Previous studies have consistently reported elevated IL-6 levels among patients with major affective disorder and ADHD.^9,32,34^ A meta-analysis of 3212 patients with MDD and 2798 healthy controls revealed significantly higher levels of IL-6 in patients than in controls.^34^ Similarly, Solmi et al. demonstrated that patients with BD had elevated IL-6 levels compared with controls, regardless of mood state.^35^ As mentioned, patients with ADHD have also consistently exhibited elevated IL-6 levels.^9^ In the current study, both first-episode BD and MDD were associated with increased IL-6 levels, whereas adolescents with ADHD only exhibited a trend toward elevated IL-6 levels.

After adjustment for clinical symptoms, we observed that adolescents with first-episode BD exhibited significant impairments in inhibitory control function, whereas those with MDD and ADHD did not. This suggests that deficits in inhibitory control may be symptom-related conditions in MDD and ADHD, whereas in BD, they may represent a biological trait.^36,37^ Lombardo et al. assessed inhibitory control function by using the self-report Barratt Impulsiveness Scale, version 11 (BIS-11) in 54 euthymic patients with BD and their healthy siblings. Their findings revealed that both the patients and their siblings exhibited significantly higher total BIS-11 scores compared with unrelated healthy controls.^36^ Similarly, Brand et al. demonstrated that patients with BD and their healthy siblings shared similar patterns of impairment in the go/no-go task.^37^ Additional studies with a large sample size of adolescents with major affective disorders and those with ADHD, as well as their healthy siblings, are warranted to validate our findings.

Several limitations of this study warrant consideration. First, we assessed inhibitory control function by using only the go/no-go task and measured only CRP, IL-6, and MMP-9 levels. Future research should investigate the relationships between different cognitive functions and other proinflammatory cytokines in adolescents with ADHD or first-episode major affective disorders, particularly in the context of SED. Second, to avoid the exacerbation of mood and behavioral symptoms, the participants continued their psychotropic medications during cognitive function assessment and biomarker measurement processes. No studies have investigated the effects of psychotropic medications on MMP-9 levels, although research has shown that certain psychotropic drugs, such as atypical antipsychotics, can have adverse effects on cytokine levels.^38^ Although this approach of maintaining medications was ethically necessary, future research employing drug-free designs is essential to validate our findings. Third, this study examined the laboratory measures (MMP-9, CRP, and IL-6) individually rather than in combination. Future research would be needed to explore whether combining these laboratory measures could be more clinically useful for identifying mental disorders and SED. Finally, regarding the multiple comparison correction for the laboratory measures, the statistical threshold for the p-value was adjusted from 0.05 to 0.017 (0.05/3). As a result, the statistical significance of the difference in MMP-9 levels between the 2 SED groups was no longer observed. Further studies with a large sample size of patients with SED would be necessary to elucidate an association between SED and MMP-9 levels.

In conclusion, adolescent psychiatric disorder diagnoses were associated with elevated levels of MMP-9, IL-6, and CRP and with inhibitory control dysfunction. Specifically, adolescents with first-episode BD exhibited the highest levels of MMP-9 and CRP and the poorest performance on the go/no-go task compared with the other groups. SED was associated with only increased MMP-9 levels, regardless of diagnostic categories. Further research is warranted to elucidate the complex associations between ADHD, first-episode major affective disorders, MMP-9, proinflammatory cytokines, and cognitive function.

## Data Availability

The datasets collected and/or analyzed during the current study are available from the corresponding author upon reasonable request. However, they are not publicly accessible due to ethical regulations governing clinical trials in Taiwan.
